# The Therapeutic Potential of MicroRNAs in Cancer: Illusion or Opportunity?

**DOI:** 10.3390/ph13120438

**Published:** 2020-12-01

**Authors:** Orazio Fortunato, Marilena V. Iorio

**Affiliations:** 1 Tumor Genomics Unit, Department of Research, Fondazione IRCCS Istituto Nazionale dei Tumori, via Venezian 1, 20133 Milan, Italy; 2 Molecular Targeting Unit, Department of Research, Fondazione IRCCS Istituto Nazionale dei Tumori, Via Amadeo 42, 20133 Milan, Italy

**Keywords:** microRNA, cancer, therapy

## Abstract

The functional involvement of microRNAs in human neoplasia has raised in the last years an increasing interest in the scientific community toward the potential application in clinics as therapeutic tools. Indeed, the possibility to modulate their expression to re-establish a lost equilibrium and counteract tumor growth and dissemination, and/or to improve responsiveness to standard therapies, is promising and fascinating. However, several issues need to be taken into account such as factors related to miRNA stability in the blood, tissue penetration and potential off-target effects, which might affect safety, tolerability and efficacy of an miRNA-based therapy. Here we describe the most relevant challenges related to miRNA-based therapy, review the delivery strategies exploited to date and the on-going clinical trials.

## 1. MiRNA Biogenesis

MiRNAs are small, naturally occurring, noncoding RNA molecules ubiquitously expressed and involved in basically any physiological process. They exert regulatory functions on gene expression by acting at the post-transcriptional level, modulating mRNA stability or affecting translation, depending on the degree of sequence complementarity with the target mRNA molecule [1].

Conserved across species, microRNAs were initially thought to be originated from junk DNA, and it took years to understand that these portions of noncoding DNA generate instead functionally active RNA molecules. Unexpectedly, the results of the human genome project demonstrated that over 98% of the human genome does not encode for proteins, revealing the existence of a huge amount of never explored information, a sort of secret book never interpreted before.

The first microRNA was described back in 1993 by Ambros and his group [2], who described the role of lin-4 in the development of the nematode *C. elegans*. However, only 7 to 8 years later these tiny RNAs had returned to the spotlight with increasing evidence of their role also in vertebrates and humans.

MiRNA biogenesis is a multistep process which leads to the production of a single-strand RNA able to bind a target messenger RNA upon loading into the RISC (RNA-induced silencing) complex. The production of a mature miRNA starts in the nucleus where a long primary transcript (pri-miRNA) is first cleaved by an enzyme complex (Drosha endoribonuclease and DGCR8/Pasha) into a double strand precursor approximately 70 nt long and characterized by the presence of a hairpin [3]. The pre-miRNA is then exported through an Exportin-5-dependent mechanism to the cytoplasm [4] where it is further cleaved by the RNAse Dicer, which removes the hairpin. The two strands of the short duplex are then opened by a helicase into two mature products, one called miR-5p, the other miR-3p, miR* or the passenger strand. Both strands are functionally active [5]. The miRtron pathway is a noncanonical process pathway which does not require the Drosha-mediated cleavage; instead it leads to pre-miR production by alternative splicing [6]. MicroRNA expression is altered in human diseases, including cancer. Although miRNAs are extensively studied, and their role in cancer was proved by several published papers, available databases probably have plenty of false positive sequences that are not derived from miRNA genes [7]. To solve this issue, a new database was created, mirGeneDB, that reviewed miRNA entries present in MiRBase and identified only one third of 1.881 miRNAs that could be considered true miRNAs [8].

## 2. MiRNA in Cancer

The involvement of microRNAs in human cancer is currently well documented. After the first evidence of an aberrant expression in neoplastic tissues versus the normal counterpart [9,10], the past 15 years have witnessed an increasing number of studies demonstrating that specific miRNA signatures associate to different tumor stages, outcome of the disease and responsiveness to specific anticancer therapies [11,12,13]. This has generated enthusiasm within the scientific community for a potential use of these molecules as biomarkers. In addition, as well known, miRNAs can be easily detected in biological fluids such as blood or serum, thus representing potential noninvasive biomarkers. Two interesting examples are the early diagnosis of lung cancer in heavy smokers [14] and the prediction of responsiveness to neoadjuvant Trastuzumab in HER2-positive breast cancer patients [15].

Nonetheless, the functional role of these tiny regulators of gene expression in human neoplasms has been clearly described. They are involved in basically all the cancer-related pathways, including proliferation, as the onco-suppressive miR-34 family [16]; migration and metastatic potential, as the oncogenic miR-9 in breast cancer [17]; stemness properties [18]; altered metabolism [19] etc. Preclinical studies demonstrating that the modulation of miRNA expression can impair a malignant phenotype, contributing to control cancer growth and dissemination and/or improving responsiveness to standard therapies, has suggested the fascinating possibility of a future miRNA-based therapy.

In lung cancer, for instance, miR-34 could be considered a key oncomiR that regulates cell-cycle, apoptosis and cellular senescence [20]. Indeed, this miRNA, finely controlled by p53, was able to modulate cell cycle genes and also to interfere with tumor growth and metastasis by directly targeting Epidermal Growth Factor Receptor (EGFR) [21]. Other miRNAs were regulated by p53 in lung cancer cells, such as miR-197 and miR-486. MiR-197 is able to control the apoptotic process in lung cancer cells [22] whereas miR-486 has a fundamental role in cancer growth as demonstrated by our and other research groups [23].

MiR-21 is over-expressed in advanced lung cancer and is considered a key regulator of cellular mechanisms such as apoptosis, proliferation and migration. Another oncogenic miRNA which promotes lung cancer proliferation is miR-17/92a cluster (mir-17, mir-18a, mir-19a, mir-20a, mir-19b, and mir-92), which is frequently over-expressed.

MiR-21 plays an oncogenic role also in breast cancer [24], whereas miR-17/92 cluster seems to have a contrasting role in different subtypes of breast cancer [25].

It has been shown that miRNAs exert a critical role in the modulation of epithelial to mesenchymal transition that leads to tumor invasion and metastasis. One of the most studied small noncoding RNAs is miR-200 which, along with miR-205, is able to modulate epithelial to mesenchymal transition (EMT) by targeting mainly the zinc finger E-box binding homeobox (ZEB1) and ZEB2 [26]. The capability of miR-205 to counteract the EMT process has been reported also in prostate cancer [27], where its function is exerted through the downregulation of protein kinase Cepsilon.

In breast cancer, we and other groups have demonstrated the oncosuppressive role of miR-205, which is able to directly target HER3, thus improving the responsiveness to tyrosin-kinase inhibitors (TKIs) [28] and Trastuzumab [29] in HER2+ breast cancer cell lines. Interestingly, it has been demonstrated that miR-205 expression is repressed by HER2 [30], plausibly a strategy exploited by the receptor to impede the inhibitory activity of the microRNA on HER3, thus maintaining a functional oncogenic dimer.

More recently, it has been become clear that microRNAs can act not only on neoplastic cells but also on components of the tumor microenvironment such as fibroblasts, endothelial cells and immune cells. It is well known that tumor cells shape a protumorigenic milieu by a birectional interaction with surrounding cells, and microRNAs have been described as key carriers of this message exchange. MiR-9, for instance, first described as oncomiR involved in the metastatization process of triple negative breast cancer (TNBC), is able to promote migration, invasion and metastasis formation [17], and has, more recently, been linked to several tumor-triggered mechanisms in the tumor microenvironment. Secreted by neoplastic cells packaged in exosomes, miR-9 is then transferred to endothelial cells [31] where it promotes cell migration by JAK/STAT pathway activation, thus favoring angiogenesis. Moreover, miR-9 can be transferred to fibroblasts where it contributes to the conversion to cancer-associated fibroblasts, which cooperate with neoplastic cells to confer a more aggressive phenotype [32].

However, miR-9 has opposite roles in different types of cancer. Whereas there is convincing evidence of an oncogenic role of this miRNA in breast cancer, its function in other tumors such as glioblastoma or uveal melanoma are more contrasting [33].

Furthermore, it has been demonstrated that the four members of the miR-181 family have a key role during embryogenesis and development of the central nervous system [34]. The MiR-181 family is also deregulated during carcinogenesis, and several studies showed modulation of fundamental cellular players such as PI3K/AKT, MAPK, TGF-β Wnt, NF-κB and Notch [35].

Recently, more comprehensive analyses considering miRNA differences at cellular levels, as performed by McCall and colleagues [36], revealed an important cell-specificity of certain miRNAs, and underlined the need for a careful evaluation of expression studies. MiR-486, for instance has been reported mainly expressed by red blood cells [37], and a differential expression between two tissues might be biased by the presence of blood cells. The evidence of an miRNA tissue specificity also provides new insights for the use of miRNAs as therapeutic agents. Studies concerning miRNA replacement for cancer treatment should be deeply analyzed using, for example, in situ miRNA hybridization in tissues [38]. In addition, whereas several miRNAs exert similar functions in different tumor types, it is not rare that the role of a specific miRNA is context-dependent, and this is an extremely important issue that needs to be considered to develop a reliable and safe miRNA-based therapy.

## 3. Clinical Trials Using MiRNAs

It is well known that miRNAs could be considered promising therapeutic agents for cancer treatment, and several pharmaceutical companies in collaboration with academic laboratories are already involved in this clinical research [39]. For systemic miRNA delivery for cancer treatment there are two types of vectors: viral and nonviral carriers. However, the viral system displayed high delivery efficiency with high toxicity and strong immunogenicity and, to date, no clinical trials using viral vectors are ongoing. Thus, nonviral carriers are considered the preferred choice for clinical studies using miRNAs, even if they have generally inefficient miRNA delivery with short efficacy. Despite the great interest raised by this field, most of the current miRNA nonviral delivery systems for cancer treatment are still in preclinical studies, and no miRNA compounds have entered into a clinical phase 3 trial. To date, just a few clinical trials considering miRNA modulation as an effective strategy for cancer treatment are ongoing (Table 1).

The first clinical trial that utilized a therapeutic intervention based on miRNA modulation was MRX34, where liposomes containing miR-34 were administered to patients with unresectable liver cancers, and other solid malignancies, to assess pharmacokinetics and pharmacodynamics. Since liposomal drugs naturally accumulate in the liver, the first indication for MRX34 was the treatment of liver cancer. The delivery vehicles were very interesting because these liposomes called Smarticles have a negative charge at normal body pH in the blood circulation, but they became cationic at lower pH, as happens in the tumor microenvironment, increasing their half-life and facilitating their uptake into tumors. Despite the safety demonstrated in the first trial [40], the administration of MRX34 in another phase 1b trial was terminated due to severe adverse immune-related effects, raising issues whether the immune responses were induced by the liposome or by the miRNA [41]. Based on these adverse effects, an extensive preclinical investigation of possible immune-related toxicity in both immunodeficient and immunocompetent mice is required to clearly demonstrate the safety of these compounds.

Nonetheless, an miRNAs mimic approach, based on miR-16 replacement to evaluate the maximum tolerated dose in patients with recurrent mesothelioma and lung cancer, is ongoing. This technique, called Targomir is based on bacterial minicells that could be used as drug delivery vehicles. Interestingly, to increase the delivery in tumor cells, these nanoparticles are modified with the addition of an anti-EGFR antibody [42]. The first published report, on 26 patients, showed one partial response and no adverse effects in the entire patient cohort [43].

The same company developed MRG-106, also known as Cobomarsen, an inhibitor of miR-155. MiR-155 is an oncomir that is highly expressed in a wide range of cancers such as leukemia, lung cancer and breast cancer [44]. Interestingly, it was demonstrated that miR-155 regulated blood cell differentiation and proliferation. Two clinical trials are ongoing to evaluate the safety and efficacy of Cobormarsen for the treatment of lymphoma and leukemia (clinicaltrial.gov: NCT02580552; NCT03713320) [45]. The first report regarding the evaluation of the safety and tolerability of Cobomarsen revealed that this compound is well tolerated in all the 43 leukemic patients, with some partial responses [46].

Guessous and colleagues described the over-expression of miR-10b in human glioblastoma and stem cell lines. In this work, after inhibition of miR-10b, they observed a strong reduction of cell proliferation, invasion and migration of glioblastoma and stem cell lines [47]. Based on the fundamental function of mir-10b, strategies using miR-10b blocking reagents have been entered into clinical trials for the treatment of glioblastoma (clinicaltrial.gov: NCT01849952). Though there is a promising antitumoral effect, the therapeutic part of this study is still preclinical and further research is needed to confirm this result.

## 4. Challenges for MiRNA Therapy

Several biological reasons are still limiting the clinical application of miRNAs for cancer management. These limitations include factors related to miRNA stability in the blood, low tissue penetration and induction of off-target effects. In this paragraph, we describe different strategies that have been undertaken to address these challenges.

### 4.1. Rapid Clearance in the Blood

MiRNA delivery is a big issue due to their rapid clearance in the blood circulation and renal excretion. MiRNA degradation in blood could be overcome by different chemical modifications of their sequences.

An interesting work by Segal and colleagues evaluated for the first time lipidic and hydrophobic modifications to the miRNA sequence to improve stability and delivery for lung cancer treatment. Different lipid conjugates were tested and organ biodistribution was analyzed, resulting, as expected, in miRNA accumulation in liver, kidney and spleen. Interestingly, an uptake by lung cancer was observed. Unfortunately, this study lacked of any analysis of potential side effects on normal cells and immune cell toxicity [48].

A different approach for a better miRNA delivery was to attach nucleic acid to a peptide with a low pH-induced transmembrane structure called pHLIP. This modification facilitated cellular uptake due to the acidity of the tumor microenvironment. In a mouse model of B cell lymphoma, tumor development was reduced by the transport of anti-miR-155 [49]. pHLIP did not show any signs of murine distress, toxicity and renal damage, with low accumulation into the livers [50].

### 4.2. Efficient Delivery

Synthetic oligonucleotides are hydrophilic with low molecular weights, so their capacity to penetrate cellular membranes is very limited [51]. To overcome these limitations, different carriers were developed and tested in several cancer models such as viral vectors, lipid-nanoparticles and aptamers. MiRNA modulation strategies using viral vectors represents an option unlikely to be applied in a clinical context due to the same issues of gene therapy related to limited infectivity, and problems in the transcription of the gene product. Furthermore, the known deficiencies in the miRNA processing machinery of cancer cells could be an important limitation for the use of a viral vector for cancer management. Lipid-nanoparticle systems could be considered one of the most promising vectors for miRNA delivery into cancer cells. Indeed, encapsulation of oligonucleotides in lipid nanoparticles protects them from nuclease degradation and increases the stability of miRNAs in the blood circulation. However, the design of liposomes is a very tricky point for the development of new therapeutic drugs based on miRNA modulation. Cationic liposomes were reported to be highly immunogenic due to the recruitment of immune cells as monocytes, and consequent production of proinflammatory cytokines [52]. Interferon responses have also been described after liposomal treatments in mouse models [53].

We previously demonstrated the deregulation of miR-660, now belonging to the miR-188 family, in lung cancer tissues [54]. Our group has developed coated cationic lipid-nanoparticles entrapping miR-660 mimics for the treatment of lung cancer patient-derived xenografts (PDXs) [55]. We clearly demonstrated tumor reduction in p53 wild type lung cancer without any acute or chronic toxic effects. Interestingly, we did not observe any signs of immune response either in immunocompetent mice after several treatments with these nanoparticles. One of the problems for the use of lipid-nanoparticles entrapping miRNAs is the low delivery in cancer cells. Therefore, to improve the efficacy of these compounds the addition of a tumor cell-specific ligand on the lipid surface could be an interesting alternative. Furthermore, targeted carriers potentially improve treatment efficacy by reducing therapeutic doses and preventing side effects in other cells.

An interesting work in neuroblastoma mouse models demonstrated that systemic delivery of miR-34a and let-7b reduced tumor growth. The authors designed cationic liposomes with aGD_2_-Fab’ fragments that are considered selective targets for neuroblastoma. In this preclinical model they demonstrated high stability and binding capacity and, more importantly, the absence of any acute or chronic toxicity nor immune-stimulation after liposomal treatments [56]. In a transgenic leukemia mouse model, it was demonstrated that miR-26 replacement was able to reduce leukemia cells. In this work, the authors developed lipid nanoparticles formulation with an anti-CD38 on their surface, which considerably increased delivery into leukemic cells. However, no acute and chronic toxicity studies were performed as miRNA was accumulated in liver and other organs [57].

Aptamers have been used in the past for the delivery of RNA ligands such as siRNA and miRNAs. Interestingly, aptamers show dual inhibitory effects through the conjugation of RNA inhibitory sequence with the delivery of chemotherapeutics cargos [58]. The same groups tested the antitumoral efficacy of this aptamer with a let-7g inhibitor in a mouse model of lung cancer [59]. Aptamers could be considered interesting therapeutic reagents due to their low cost of production, high facility of modification, no unwanted induction of immune response and high tissue penetration. However, aptamers could also be useful in targeting immune cells, suggesting an interesting alternate therapeutic option to antibodies. These molecules could be used as diagnostic tools for the detection of immune status, whereas they also induced protective immunity against cancer. Aptamers could be also used in immune-modulation therapy by blocking the inactivating activity of cytotoxic T-cell antigen 4 (CTLA-4) on T cells [60] or by inhibiting the function and phenotypes of tumor-associated macrophages [61].

### 4.3. Reduction of off-Target Effects

Since miRNAs are able to simultaneously target multiple pathways in different cells, they potentially affect the levels of tumor suppressor genes in normal cells. One of the most studied miRNAs in lung is let-7, which is highly expressed in normal lung tissue. This miRNA regulates directly several genes related to cellular proliferation, such as KRAS and NRAS. Let-7 is able to repress in lung cells other tumor suppressor genes such as BRCA1, BRCA2 or cell cycle checkpoints such as MAD2L1 and CDC23 [62]. In small cell lung cancer, miR-335 is aberrantly expressed in metastatic cells with skeletal bone tropism. This miRNA is able to directly modulate the expression levels of RANK ligand (RANKL) and insulin-like growth factor-1receptor (IGF-1R), that are known mediators of bone metastases [63].

One of the biggest issues regarding the use of miRNAs as therapeutic compounds is the reduction of this off-target effect that may cause potential toxicities and reduced therapeutic effects.

MiRNAs duplexes have been demonstrated to be important inducers of immune cell activation with the release of pr-inflammatory cytokines and interferons. The activation of innate immunity happens through direct interactions with Toll-Like Receptors (TLRs) in a similar way to viral and bacterial RNA and DNA [64]. Indeed different TLRs, including TLR3, TLR7, TLR8, and TLR9, were able to recognize nucleic acids with the activation of different cells such as macrophages [65]. MiR-21 and miR-29 in exosomes from lung cancer cells were able to bind directly TLR8 expressed by macrophages, and induced the release of proinflammatory cytokines such as interleukin-6 and tumor necrosis factor-α [66]. Furthermore, several studies reported miRNAs as regulators of TLR expression and activity on immune cells. Since some of these miRNAs, such as let-7, miR-26a, miR-223 and miR-511, are involved in cancer development and progression [67], the potential activation of TLR signaling should be considered and tested in preclinical studies. The interactions of miRNAs-TLRs potentially stimulate the innate immune cells, resulting in severe toxicity and adverse effects. One of the most important issues for miRNAs as therapy is to avoid the activation of immune systems through the engagement of TLRs that lead to proinflammatory responses in the host.

## 5. New Frontiers: Extracellular Vesicles as Vehicle for MiRNA Delivery

Extracellular vesicles (EVs) are an evolutionarily conserved group of bilayer membrane vesicles [68,69] generally classified by size and intracellular origin into two main categories: small EVs (sEVs, also called exosomes) derived from multivesicular bodies of late endosomes (~50–150 nm in diameter), and microvesicles (MVs or ectosomes) generated via extracellular membrane budding (~100 nm–1 mm in diameter) [70].

Initially described as cellular garbage for the elimination of unwanted molecules from cells, now they have been described with a key role in cell-to-cell communication both in normal and pathological states. EVs contain several biomolecules, such as lipids, proteins and nucleic acids (DNA, mRNAs, long noncoding RNAs and miRNAs) that can be shuttled to distant cells and influence the phenotype and the function of recipient cells. It has been shown that EVs are closely related to carcinogenesis, and that tumor-derived EVs exert an important role in cancer growth and progression, modulating a wide range of pathways including tumor angiogenesis and EMT [71]. Fabbri and colleagues demonstrated that exosomal-miR-21 and miR-29a released by lung tumor cells are able to bind TLR on the surface of immune cells, leading to an activation of proinflammatory pathways that support lung tumor growth and metastasis [66]. Secretion of vesicles with high levels of miR-122 by breast cancer cells was able to suppress glucose uptake by nontumor cells during the formation of the premetastatic niche. This study clearly demonstrated that tumor-derived EV and their miRNAs content could reprogram glucose metabolism to facilitate metastatic dissemination [72]. It has been described that macrophages released miR-223 in microvesicles to support breast cancer cell growth and invasiveness [73], whereas miR-23 from bladder cancer EVs cancer actively promoted cancer metastatization [74]. In addition, miR-103a from hypoxic lung cancer cells induced an M2 macrophage phenotype through AKT and STAT3 activation that led to tumor progression and angiogenesis [75].

The use of EV from cancer patients as carriers for miRNA delivery may potentially represent a biocompatible and safe tool in clinical applications [76]. The therapeutic potential of EVs in cancer has been evaluated in clinical trials to demonstrate safety and antitumoral efficacy. Indeed, a phase II trial using dendritic cell-derived exosomes loaded with MAGE (melanoma associated antigen) peptides to treat unresectable nonsmall cell lung cancer is still ongoing (clinicaltrials.gov/NCT01159288) [77]. Starting from a published work that revealed exosomes as an efficient vehicle for curcumin as an anti-inflammatory agent into malignant colon cancer cells [78], a phase I clinical trial investigating the ability of these exosomes to deliver curcumin to colon cancer tissue is still active (clinicaltrials.gov/NCT01294072).

Compared with nanoparticles, EVs could be potentially useful carriers for miRNA delivery, increasing biodistribution and reducing nontargeted cytotoxicity and immunogenicity of synthetic oligonucleotides. Additionally, compared with other compounds, EV-based delivery could greatly reduce side effects in normal tissues. Recent studies have demonstrated that the cargo of EVs could be altered by adding synthetic oligonucleotides. For example, miRNAs can be easily loaded into EVs by direct transfection of miRNAs’ mimics or inhibitors. For instance, miR-21 sponges were inserted into exosomes and demonstrated the antitumoral effects of these compounds in a rat model of glioblastoma. After single administration of engineered exosomes, the authors observed tumor inhibition with modulation of miR-21 targets at 18 days [79]. An interesting work illustrated the modification of the exosomal surface and cargo. Indeed, the authors added on their vesicles a peptide targeting EGFR on cancer cells that increased the specific delivery to cancer tissues after intravenous injection of exosomes in mice. Synthetic let-7a was entrapped in these EVs and was able to reduce tumor growth in RAG2^−/−^ mice without any signs of damage in mouse organs [80]. Several efforts should be made to create factories, as already done for cell therapy, in which EVs could be produced from patients’ cells previously modified to internalize specific therapeutic molecules. This may reduce production cost and, more importantly, the potential side effects of these treatments for cancer. To successfully translate EVs into the clinic, the main problem is to efficiently scale up the process for clinical use. Indeed, to date there is a lack of reproducible methods for the generation of large batches of EVs isolated from a single source, such as cells or biofluids.

Although there is great expectation for the use of EVs as diagnostic or therapeutic tools for cancer management, several efforts should be made to overcome problems such as EV production, endocytosis and mechanism of action.

## 6. Conclusions

Several issues need to be taken into account for a concrete application in clinics of miRNA modulation, such as miRNA stability and efficient delivery, as well as potential side effects and consequent toxicity.

**Figure 1:**
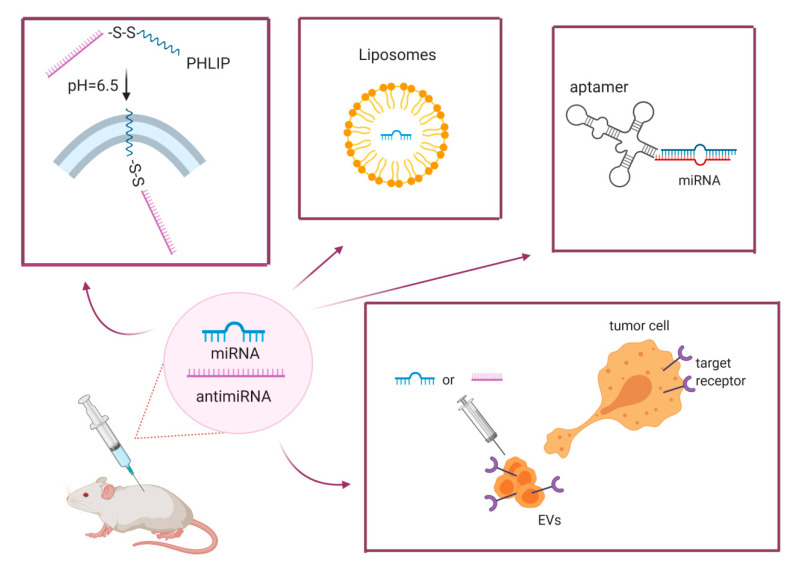
Graphical representation of miRNA/anti-miRNA delivery strategies.

Several approaches have been tested to overcome these limitations and optimize delivery, including chemically modified oligonucleotides, lipidic carriers, viral vectors and, more recently, promising EVs, which can also be engineered to increase tumor-specific delivery, thus improving efficiency and limiting toxicity (Figure 1).

Despite the still open questions, and the need to find the optimal strategy to balance the efficacy and the safety of an miRNA-based therapy, the on-going trials and the novel preclinical evidence are promising.

The cartoon summarizes some of approaches developed to deliver miRNA/anti-miRNAs in vivo, including the PHLIP system where the uptake of the miRNA-carrying complex is triggered by a change in the pH; aptamers; liposomes, and the more recently described extracellular vesicles, which can be loaded with the miRNA/anti-miRNA of interest and modified to obtain tumor-specific delivery.

## Figures and Tables

**Table 1:** Clinical trials using miRNAs as therapeutic targets.

Therapeutic Agent	Vectors	Disease	Clinical Trial Phase	Status
miR-34 mimic (MRX34)	Smarticles	Liver and solid cancer	Phase I (NCT01829971)	Terminated
miR-16 mimic (MesomiR-1)	Targomir	NSCLC	Phase I (NCT02369198)	Completed
anti-miR-155 (Cobomarsen)	locked nucleic acid	lymphoma and leukemia	Phase II (NCT02580552; NCT03713320	Active, not recruiting
anti-miR-10b	miRNA inhibitor	glioblastoma	Phase I (NCT01849952)	Recruiting
